# EFFECTIVENESS OF LIGAMENTUM TERES CARDIOPEXY FOR GERD RESOLUTION AFTER SLEEVE GASTRECTOMY: A META-ANALYSIS AND SYSTEMATIC REVIEW

**DOI:** 10.1590/S0004-2803.24612025-080

**Published:** 2026-03-23

**Authors:** Pedro Bicudo BREGION, Victor Kenzo IVANO, Luca Maunsell PEREIRA, Isadora CHRISPIM, Everton CAZZO, Anna Carolina Batista DANTAS

**Affiliations:** 1 Universidade Estadual de Campinas (UNICAMP), Faculdade de Ciências Médicas, Estudante de Graduação, Campinas, SP, Brasil.; 2 Universidade Estadual de Campinas (UNICAMP), Pesquisador Colaborador, Departamento de Cirurgia, Campinas, SP, Brasil.; 3 Universidade Estadual de Campinas (UNICAMP), Professor Associado, Departamento de Cirurgia, Campinas, SP, Brasil.; 4 Hospital Universitário Onofre Lopes, Departamento de Cirurgia, Professor Assistente, Petrópolis, Natal, RN, Brasil.

**Keywords:** Gastroesophageal reflux, gastrectomy, bariatric surgery, cardioplasty, round ligament of liver, Refluxo gastroesofágico, gastrectomia, cirurgia bariátrica, cardioplastia, ligamento redondo do fígado

## Abstract

**Background:**

Gastroesophageal Reflux Disease (GERD) is the most common long-term complication after Sleeve Gastrectomy (SG). Ligamentum teres cardiopexy (LTC) has been proposed as a revisional alternative to treat GERD symptoms.

**Objective:**

To evaluate, through a systematic review and meta-analysis, the safety and effectiveness of LTC in the remission of GERD symptoms and in reducing the use of anti-reflux medications in patients who previously underwent SG.

**Methods:**

We conducted a systematic review and meta-analysis to evaluate LTC’s safety and its impact on GERD after SG. We systematically searched PubMed, EMBASE, and Cochrane Central for studies assessing LTC in patients who underwent SG up to april 2025. The primary outcomes included remission of GERD symptoms and prevalence of GERD medications after surgery. Secondary outcomes were length of stay and adverse effects. Observational studies were included. A random-effects model analysis for GERD remission and prevalence of GERD medications were performed. Study weights were calculated using the inverse variance method, with statistical analyses conducted in R version 4.4.0.

**Results:**

Four studies including 193 patients were analyzed. Overall, remission of GERD symptoms among patients undergoing revisional LTC alone, the remission rate was 75.50% (95%CI 59.55-86.57; I²=41.4%). Additionally, 33.42% of patients (95%CI 19.74-50.60; I²=44.7%) needed to use GERD-related medication postoperatively.

**Conclusion:**

LTC shows potential as a surgical option for managing GERD after SG, particularly in revisional cases as an alternative to Roux-en-Y gastric bypass.

## HIGHLIGHTS

• Ligamentum teres cardiopexy (LTC) may serve as a viable surgical option for patients with persistent GERD after sleeve gastrectomy, especially those who are not suitable candidates for conversion to Roux-en-Y gastric bypass.

• Current evidence does not support the routine use of LTC as a primary anti-reflux intervention during initial sleeve gastrectomy due to limited supporting data.

• Surgeons should avoid over-tightening the valve during LTC to prevent postoperative dysphagia, and more robust, randomized studies are needed to better define LTC’s role in both primary and revisional bariatric surgery.

## RESUMO

**Contexto:**

A Doença do Refluxo Gastroesofágico (DRGE) é a complicação de longo prazo mais comum após a gastrectomia vertical (GV). A cardiopexia com ligamento redondo (ligamentum teres) (LTC) tem sido proposta como uma alternativa revisional para tratar sintomas de DRGE.

**Objetivo:**

Avaliar, por meio de revisão sistemática e meta-análise, a segurança e a efetividade da LTC na remissão dos sintomas de DRGE e na redução do uso de medicações anti-refluxo em pacientes previamente submetidos à GV.

**Métodos:**

Conduzimos uma revisão sistemática e meta-análise para avaliar a segurança da LTC e seu impacto na DRGE após GV. Realizamos uma busca sistemática nas bases PubMed, EMBASE e Cochrane Central por estudos que avaliaram a LTC em pacientes submetidos à GV até abril de 2025. Os desfechos primários incluíram remissão dos sintomas de DRGE e prevalência do uso de medicações para DRGE após a cirurgia. Os desfechos secundários foram tempo de internação e efeitos adversos. Estudos observacionais foram incluídos. Uma análise de modelo de efeitos aleatórios para remissão da DRGE e prevalência do uso de medicamentos foi realizada. Os pesos dos estudos foram calculados pelo método de variância inversa, com análises estatísticas conduzidas no R versão 4.4.0.

**Resultados:**

Quatro estudos, totalizando 193 pacientes, foram analisados. No geral, entre os pacientes submetidos à LTC revisional isolada, a taxa de remissão dos sintomas de DRGE foi de 75,50% (IC95% 59,55-86,57; I²=41,4%). Além disso, 33,42% dos pacientes (IC95% 19,74-50,60; I²=44,7%) necessitaram usar medicamentos relacionados à DRGE no pós-operatório.

**Conclusão:**

A LTC demonstra potencial como opção cirúrgica para o manejo da DRGE após GV, especialmente em casos revisionais como alternativa ao *bypass* gástrico em Y-de-Roux.

## INTRODUCTION

Sleeve Gastrectomy (SG) is the most commonly performed metabolic and bariatric surgery (MBS) worldwide^1^. It offers sustained long-term weight loss outcomes and remission of associated clinical conditions^2^.

Despite these advantages, a growing body of evidence indicates that SG is associated with an increased risk of gastroesophageal reflux disease (GERD), erosive esophagitis, and even the development of Barrett’s esophagus^2^ ^-^ ^4^.

Post-SG GERD is typically managed with continuous pharmacological therapy, primarily alleviating symptoms of heartburn^5^. However, liquid regurgitation and atypical symptoms often show limited response to proton pump inhibitors (PPIs)^6^, significantly affecting patients’ quality of life^7^. This includes disruptions in sleep quality, limitations in physical activity, and psychological distress linked to food aversion. In the United States, gastroesophageal reflux disease (GERD) accounts for approximately 50% of revisional SG cases converted to Roux-en-Y gastric bypass (RYGB), contributing to increased healthcare costs and patient morbidity^8^.

The gold standard for managing refractory gastroesophageal reflux disease (GERD) following sleeve gastrectomy (SG) is conversion to Roux-en-Y gastric bypass (RYGB). However, not all patients are suitable candidates for revisional surgery, and some may decline the procedure. Given the need for alternative solutions that can address GERD without recurring to conversional bariatric procedures, various approaches have been proposed. These include the use of magnetic rings around the lower esophageal sphincter (LES), and LTC^9^ ^-^ ^11^. Cardiopexy, first described by Rampal in 1964, offers a novel approach to managing reflux in SG patients by restoring the intra-abdominal positioning of the LES and recreating the physiological angle of His^12^ ^-^ ^14^. While early studies have demonstrated its potential effectiveness, they are limited by small sample sizes and short follow-up periods^15^ ^-^ ^21^.

To provide a more comprehensive evaluation of ligamentum teres cardiopexy (LTC) as an anti-reflux procedure following SG as a revisional procedure, we conducted a systematic review. This analysis aims to assess the efficacy of this technique in reducing GERD symptoms and medication dependence.

## METHODS

The review has been registered with the National Institute for Health Research International Registry of Systematic Reviews (PROSPERO, CRD42024602862).

### Literature search

We performed a comprehensive literature search using PubMed, The Cochrane Library, and Embase databases, from inception to September 2024, to identify studies evaluating outcomes of SG with ligamentum teres cardiopexy. The search strategy for PubMed was ((sleeve gastrectomy) AND (“cardiopexy” OR “teres” OR “ligamentum”)). A similar search strategy was used for other databases. The study was registered in the International Prospective Register of Systematic Reviews-University of York (PROSPERO) with Registry Number CRD42024602862.

### Study selection

The study selection process followed the Preferred Reporting Items for Systematic Reviews and Meta-Analyses (PRISMA) guidelines^22^. Duplicate records were removed before two independent reviewers (PB and VK) screened the titles and abstracts of studies. Conflicts were resolved by a third reviewer (EC). Full-text articles of potentially eligible studies were retrieved for detailed evaluation based on predefined inclusion and exclusion criteria.

### Eligibility criteria

We included articles reporting on SG with ligamentum teres cardiopexy in adult patients (aged 18-70 years). The included studies reported outcomes on GERD resolution, proton pump inhibitor (PPI) therapy discontinuation, operative time, hiatal hernia status, and hospital stay duration. Study types consisted of case series and retrospective studies. We excluded non-English publications, studies lacking relevant outcome data, abstracts and case reports. The quality of the included studies was assessed using the ROBINS-I v2.

### Data extraction

Data extraction was independently performed by two reviewers (PB and VK) using Excel spreadsheets, with a third reviewer (EC) verifying the accuracy. Extracted data included study characteristics (publication year, sample size and study design), patient demographics (age, gender distribution, BMI before SG and LTC), and clinical outcomes (GERD symptoms, PPI therapy, hiatal hernia, and hospital stay duration).

### Outcomes

The primary outcome was the postoperative resolution of GERD symptoms, defined as the remission of symptoms. Secondary outcomes included the prevalence of postoperative medication. GERD resolution was analyzed in patients undergoing revisional LTC. Medication use was evaluated based on the discontinuation of proton pump inhibitors (PPIs) and other GERD-related drugs.

### Statistical analysis

Meta-analyses were conducted to pool the outcomes of interest from included studies. For binary outcomes (GERD resolution, prevalance of medication use post-operatory), proportions with 95% confidence intervals (CI) were calculated. Heterogeneity among studies was evaluated using the Cochran Q statistic and the I² statistic, with high heterogeneity defined as a *P*-value of less than 0.05 and an I² greater than 50%. A random-effects model was employed to account for between-study variability.

To assess the robustness of the results, we performed leave-one-out sensitivity analyses. Funnel plots and Egger’s test were used to evaluate publication bias for each outcome. All statistical analyses were conducted using R (version 4.4.2, R Project for Statistical Computing).

Some studies have evaluated ligamentum teres cardiopexy (LTC) as a primary procedure alongside sleeve gastrectomy (SG), while others have assessed it as a revisional surgery for GERD following SG. As a result, we decided to evaluate only as a revisional procedure. Primary SG with LTC are only described without quantitative analysis.

## RESULTS

### Study characteristics

**FIGURE 1:**
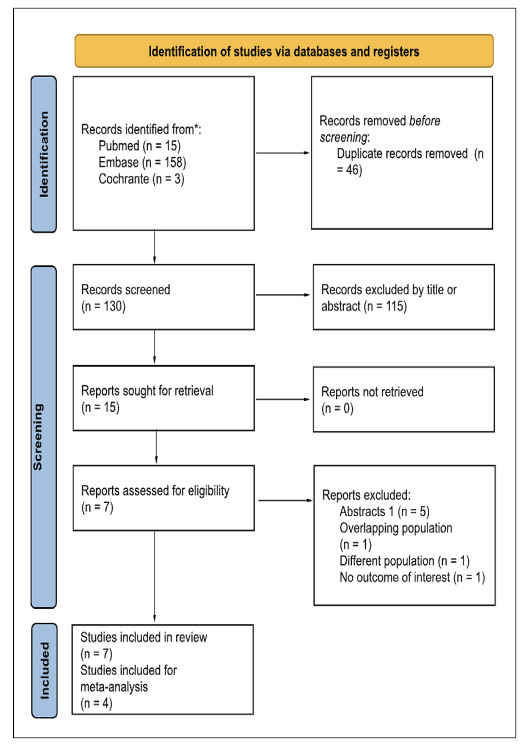
PRISMA flow diagram of study screening and selection.

The systematic search identified 176 studies. After removing 46 duplicates, 115 studies were excluded by title and abstract screening, leaving 15 studies for full-text evaluation. As shown in the PRISMA flowchart (Figure 1), seven studies^9^ ^,^ ^15^ ^,^ ^16^ ^,^ ^18^ ^-^ ^20^ ^,^ ^23^ satisfied the eligibility criteria.

### Patient characteristics

Upon aggregating data from four of the included studies as revisional LTC, the meta-analysis comprised 193 patients, with participants per study ranging from 10 to 79. The participants’ ages ranged from 35.6 to 52.9 years, and the proportion of female patients varied between 85% and 100%. BMI Pre-LTC ranged from 21.9 to 36.9 kg/m². A detailed summary of each study type, sample size and mains demographic and clinical characteristics can be found in Table 1.

**TABLE 1:** Summary of included articles.

Article	Gálvez-Valdovinos, 2015	Hawasli, 2021	Lind, 2024	Mackey, 2023
N of patients	15	10	29	60
Type of Study	Case series	Case series	Retrospective chart review	Retrospective chart review
Type os Surgery	Revisional LTC	Revisional LTC	Revisional LTC	Revisional LTC
Age (mean±sd)	35.6±15.2	52.9±8.4	50±9.1	48±3.7
Gender (Female %)	86.7	100	86.2	85
BMI Pre-LTC (mean±sd)	21.9±2.2	30.7±3.9	36.1±5.4	36.9±1.8
Interval (months) since SG	30	NR	59.9±34.9	34.8 (26.1 - 56.4)
pre operative GERD Symptoms (Yes %)	100	100	100	100
pre LTC esophagitis (Yes%)	100	50	NR	NR
post LTC esophagitis (Yes%)	13.3	NR	NR	NR
pre LTC PPI Therapy (Yes %)	100	100	100	100
post LTC PPI (Yes%)	86.6	20	42.9	44
Hiatal Hernia (Yes %)	100	NR	NR	NR
Hospital Stay Duration (mean±sd)	NR	NR	1.3±0.3	NR
Median follow-up (months)	6	7±3	17.3±11.1	12

BMI Pre-LTC: body mass index before ligamentum teres cardiopexy (LTC). GERD: gastroesophageal reflux disease. NR: not reported. PPI: proton pump inhibitor therapy. sd: standard deviation. SG: sleeve gastrectomy. DJB: duodenojejunal by-pass. PJB: proximal jejunal by-pass.

GERD symptoms were highly prevalent, with up to 100% of patients affected in revisional cases. Esophagitis was observed in 50-100% of cases, and 100% of patients required PPI therapy before LTC. All patients requiring hiatal hernia repair had the procedure performed concurrently with LTC, which may have influenced the postoperative outcomes. Follow-up was short with a median range of 6 to 30 months.

### Meta-analysis for revisional LTC for refractory GERD after SG - quantitative data

Caballero et al. and Huang et al. were excluded from the meta-analysis as they do not directly address the core research question. In both studies, it is unclear whether the observed GERD resolution was attributable to weight loss or to the ligamentum teres cardiopexy (LTC) itself. In the absence of a comparative design-such as that used in Moon’s study, which analyzed similar populations with and without LTC-the isolated effect of LTC cannot be reliably assessed. Moreover, the expected resolution rate tends to be lower when LTC is performed alongside sleeve gastrectomy, since these patients often present with preexisting GERD and potential underlying motility disorders. Conversely, patients undergoing revisional cardiopexy typically develop GERD as a consequence of sleeve gastrectomy, which is a more straightforward condition potentially corrected through surgical revision alone. In addition, Furthermore, in Huang et al., nearly half of the patients had a concomitant bypass procedure, which makes this study unique compared to others.

### Resolution of GERD after surgery

**FIGURE 2:**
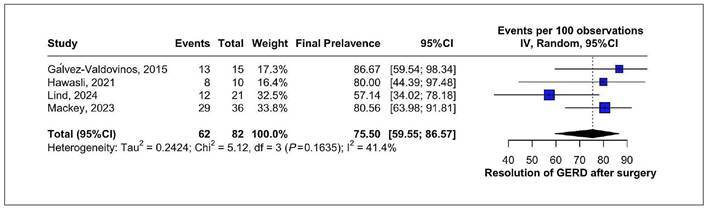
Resolution of GERD after LTC surgery.

**FIGURE 3:**
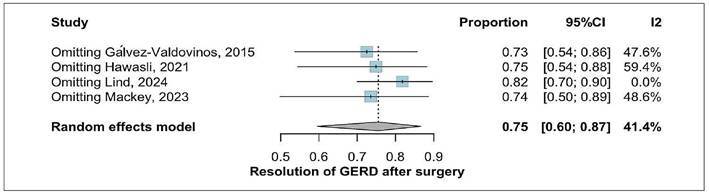
Leave-one-out analysis of GERD resolution.

One forest plot was created to summarize the resolution of GERD symptoms following LTC as a revisional procedure. The analysis included 4 studies, with a total of 82 patients. GERD resolution rate was 75.50 % (95%CI: 59.55-86.57) with moderate heterogeneity I²=41.4%. Figure 2 presents the forest plot summarizing these findings. A leave-one-out sensitivity analysis was conducted due to the visual appearance of Lind’s study as a potential outlier. Omitting this study reduced heterogeneity to 0% and resulted in only a minimal change in the pooled GERD resolution rate to 82% (95%CI: 70-90%), thereby reinforcing the robustness of the overall findings (Figure 3).

### Prevalence of medication use after surgery

**FIGURE 4:**
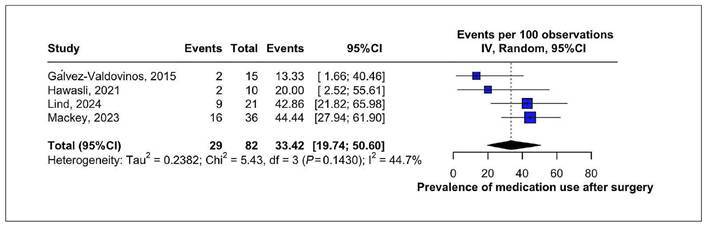
Prevalence of GERD medication use after surgery.

**FIGURE 5:**
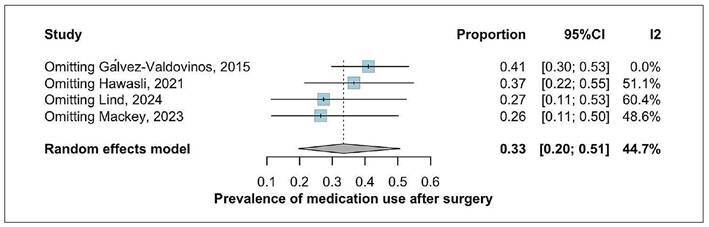
Leave-one-out analysis of GERD medication after surgery.

Overall, postoperative medication use was reported in four studies comprising 82 patients. The pooled prevalence of continued GERD-related medication use was 33.42% (95%CI: 19.74-50.60), with moderate heterogeneity (I²=44.7%) (Figure 4). A leave-one-out sensitivity analysis identified the study by Gálvez-Valdovinos as a potential outlier. Its exclusion reduced heterogeneity to 0% and slightly increased the pooled prevalence to 41% (95%CI: 30-50%). Gálvez-Valdovinos et al. optimistic results may be explained by its small sample size, potential selection bias, and the fact that it represented one of the earliest reports on the technique within a larger series (Figure 5).

### Length of stay

**FIGURE 6:**
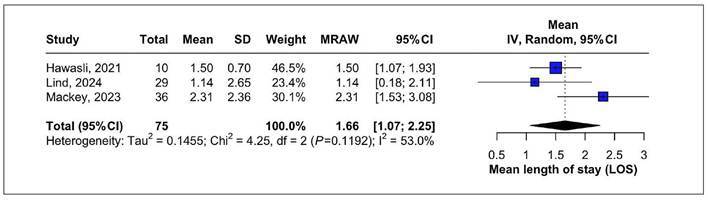
Mean length of stay in days.

**FIGURE 7:**
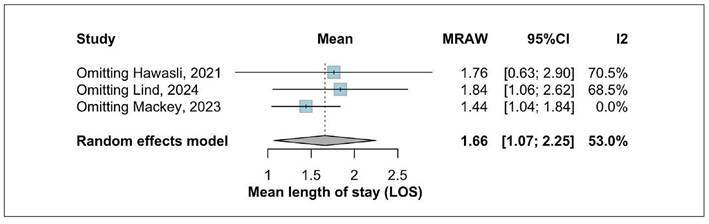
Leave-one-out of length of stay.

One forest plot was created to summarize the length of stay after LTC. The analysis included 3 studies, with a total of 75 patients. Length of stay was 1.66 days (95%CI: 1.07-2.25) with moderate heterogeneity I²=53% (Figure 6). A leave-one-out sensitivity analysis was conducted due to the visual appearance of Mackey’s study as a potential outlier. Omitting this study reduced heterogeneity to 0% and resulted in only a minimal change in the pooled length of stay resolution rate to 1.44 (95%CI 1.04-1.84), thereby reinforcing the robustness of the overall findings (Figure 7).

### Adverse effects

**FIGURE 8:**
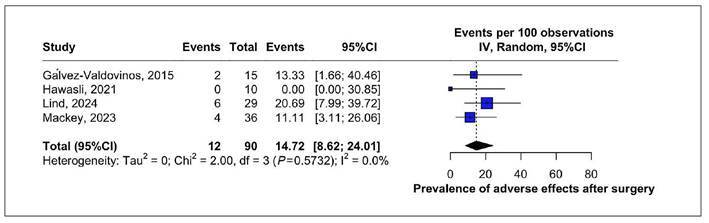
Prevalence of adverse effects after revisional LTC.

A forest plot was generated to assess the prevalence of adverse events following revisional LTC, revealing a pooled rate of 14.72% (95%CI: 8.62-24.01) with low heterogeneity (I²=0%). These complications, detailed in the systematic review, were predominantly minor in nature (Figure 8).

## SYSTEMATIC REVIEW - Narrative data

### Surgical techniques

The surgical approaches varied considerably across the seven studies included in the review. Both primary and revisional ligamentum teres cardiopexy (LTC) were performed, often in the context of sleeve gastrectomy (SG). While some authors-such as Caballero and Moon-described LTC in conjunction with primary SG, the majority of studies focused on revisional procedures addressing persistent or recurrent GERD. The use of adjunct procedures like jejunal or duodenojejunal bypass, particularly in Huang’s study, which presented a hybrid approach.

### Indications for LTC

Revisional LTC was consistently reserved for patients with refractory GERD who declined conversion to Roux-en-Y gastric bypass (RYGB), indicating its role as a sphincter-preserving alternative. Across studies, patients typically presented with GERD symptoms unresponsive to prolonged proton pump inhibitor (PPI) therapy. The narrative reveals that LTC served as a compromise for patients unwilling to undergo more extensive surgical revision.

### Surgical valve details

The technique of valve reconstruction varied, with most authors utilizing either a 270° or a complete 360° posterior wrap using the ligamentum teres. The use of hiatal hernia repair and calibration tubes (e.g., 36 Fr) was inconsistently reported, suggesting potential heterogeneity in technical execution. While Moon did not detail the technique, other authors highlighted the importance of individualized valve construction based on patient anatomy and disease characteristics.

### Length of Stay

Postoperative hospital stay was generally short across all studies, with most patients discharged within 1 to 3 days. Interestingly, Moon reported a significantly shorter stay in the SG + LTC group compared to SG alone, possibly reflecting a protective effect of the cardiopexy on early postoperative recovery.

### GERD outcomes and recurrence

Symptom resolution rates after LTC were promising but variable. Complete GERD resolution was frequently observed within the first 6 to 12 months; however, recurrence was not uncommon. Caballero reported early success followed by high recurrence, leading to study discontinuation. In contrast, Galvez-Valdovinos, Hawasli, and Huang reported durable outcomes in most patients. Notably, Lind and Mackey observed partial responses, with some patients requiring continued medical therapy or even RYGB conversion. This suggests that while LTC may offer relief in a majority of cases, a subset of patients with complex motility disorders or anatomical abnormalities may remain refractory.

### Endoscopy and manometry findings

Postoperative evaluation using endoscopy and manometry was inconsistently reported but offered insights into the functional effects of LTC. LES pressure elevation was noted by Caballero, suggesting increased barrier competence. Nonetheless, anatomical failures such as recurrent hiatal hernia or esophagitis were observed in a few patients, reinforcing the need for careful patient selection and technique standardization.

### Prevalence of medication use after surgery

Despite symptom improvement, a notable proportion of patients continued PPI therapy long term. Caballero, Lind, and Mackey all reported that nearly one-third to half of their cohorts remained on antisecretory medications, reflecting either incomplete resolution or recurrence of GERD. In contrast, Galvez-Valdovinos’ study stood out with minimal reliance on PPIs postoperatively.

### Operative time

Operative duration varied widely, ranging from 60 to over 110 minutes. The longer operative times in Hawasli’s series may reflect the technical demands of the 360° wrap or case complexity in revision settings.

### Complications

Overall, LTC was associated with a low incidence of severe complications. Most adverse events were minor or managed nonoperatively, such as dysphagia requiring endoscopic dilation or transient chest discomfort. However, a small proportion of patients required surgical reintervention, including conversion to RYGB for unresolved GERD or technical failure. Notably, Moon’s series reported no complications, which may reflect differences in patient selection or operative technique.

## DISCUSSION

This meta-analysis evaluated the role of ligamentum teres cardiopexy (LTC) in the management of refractory gastroesophageal reflux disease (GERD) following sleeve gastrectomy (SG), with a focus on GERD resolution and postoperative medication use. The findings demonstrated a high short-term rate of symptom resolution after LTC with a brief hospital stay; however, a considerable proportion of patients continued to require GERD-related pharmacological therapy postoperatively. Minor complications were observed but were predominantly managed conservatively. Collectively, these results suggest that LTC may represent a feasible and effective alternative for patients with persistent GERD after SG who are either unwilling or unsuitable for standard surgical conversion to Roux-en-Y gastric bypass (RYGB).

LTC builds on existing gastropexy techniques, such as those using the greater omentum or gastrocolic ligament, which have been shown to reduce gastric twisting, bleeding, and fistula rates, with a modest decrease in GERD prevalence^24^. However, the ligamentum teres offers a unique advantage in that it preserves the angle of His, a crucial component of the anti-reflux mechanism. By acting as a dynamic “floating anchor,” the ligamentum teres maintains the gastroesophageal junction (GEJ) in its optimal position, preventing intrathoracic migration and reducing reflux (FIGURE 9)^24^ ^,^ ^25^.

Initially described by Rampal in 1964, LTC was adapted from techniques aimed at reinforcing the LES without the risks associated with traditional fundoplication^12^. The resurgence of LTC in bariatric surgery highlights its role in addressing the anatomical challenges posed by SG^12^ ^,^ ^13^. LTC has also been used in conjunction with other bariatric procedures such as RYGB and One-Anastomosis Gastric Bypass (OAGB), where it helps manage reflux symptoms^14^. Acid reflux is more common after SG, while bile reflux predominates following OAGB, and reflux is rarely observed after RYGB, underscoring the need for tailored interventions^14^ ^,^ ^23^ ^,^ ^26^.

Our findings are consistent with studies by Mackey et al.^9^, who reported significant reductions in GERD symptoms and medication use after LTC in patients with refractory reflux following SG. 

Moon et al.^23^ demonstrated the feasibility of combining LTC with SG in a one-step procedure, although short-term outcomes did not show significant improvements in GERD resolution. The physiological success of LTC lies in restoring the angle of His and increasing LES pressure, which prevents reflux without the complications seen in more invasive techniques like 360-degree fundoplication^9^ ^,^ ^15^ ^,^ ^19^. Further studies are warranted to evaluate the applicability of this technique in the context of primary sleeve gastrectomy to prevent postoperative GERD. 

Reflux mechanisms after sleeve gastrectomy are still not fully understood. The “low-volume, high-pressure” environment created by SG can exacerbate reflux through LES dysfunction and altered antral motility^27^ ^,^ ^28^. Mechanical factors such as sleeve stenosis, twisting, and residual fundus contribute to GERD^14^ ^,^ ^29^. LTC addresses these issues by repositioning the GEJ, reinforcing the anti-reflux barrier through crural approximation, and restoring intra-abdominal pressure^27^. However, as shown in Caballero et al.^16^ study, GERD recurrence can still occur, possibly due to the absence of the gastric fundus, which impairs the esophagogastric valve mechanism. A randomized radiological study demonstrated a slower passage of contrast with a “bird beak” appearance, without evidence of reflux or hiatal hernia, confirming the anatomical changes achieved by LTC in maintaining the gastroesophageal junction position and reinforcing the anti-reflux barrier.

The variability in operative time across studies is worth noting, likely due to differences in surgical technique, hiatal hernia repair, and surgeon experience^9^ ^,^ ^15^. While some reported times as short as 45 minutes, others exceeded 100 minutes, suggesting the need for procedural standardization^19^. Furthermore, avoiding a 360-degree wrap, which can cause esophageal obstruction, has been emphasized, with studies advocating for a 270-degree wrap to maintain the anti-reflux effect without excessive restriction^9^ ^,^ ^14^ ^,^ ^30^.

This study has limitations. The heterogeneity of the included studies, both in methodology and patient populations, may limit the generalizability of our findings. Additionally, many included studies were observational, potentially introducing bias in GERD assessment. The subjective assessment of reflux presents a notable limitation. Ideally, objective evaluations through endoscopy and pH monitoring would provide a clearer understanding of the intervention’s impact. However, pH monitoring is often uncomfortable for patients, with poor tolerability and low adherence to sequential testing, limiting its widespread application in postoperative follow-up. Lastly, long-term follow-up and comparative data with a control group are lacking, so conclusions on the durability of GERD resolution post-LTC should be interpreted cautiously. Future studies should focus on long-term outcomes to validate LTC’s efficacy.

## CONCLUSION

Our meta-analysis suggests that ligamentum teres cardiopexy (LTC) may represent a potential surgical alternative for managing refractory gastroesophageal reflux disease (GERD) following sleeve gastrectomy (SG), particularly among those who either decline or are not candidates for standard interventions such as conversion to Roux-en-Y gastric bypass (RYGB). However, current evidence remains insufficient to recommend its routine application as a primary procedure during SG. Care should be taken to avoid excessive tightening of the valve during LTC, as this may predispose patients to postoperative dysphagia. Further high-quality, randomized studies are necessary to establish the optimal role of LTC in primary and revisional bariatric procedures.

## Data Availability

data-available-upon-request
